# One- vs. Three-Fraction Pancreatic Stereotactic Body Radiation Therapy for Pancreatic Carcinoma: Single Institution Retrospective Review

**DOI:** 10.3389/fonc.2017.00272

**Published:** 2017-11-14

**Authors:** Philip Anthony Sutera, Mark E. Bernard, Beant S. Gill, Kamran K. Harper, Kimmen Quan, Nathan Bahary, Steven A. Burton, Herbert Zeh, Dwight E. Heron

**Affiliations:** ^1^Department of Radiation Oncology, Hillman Cancer Center, Pittsburgh, PA, United States; ^2^Department of Radiation Medicine, University of Kentucky, Lexington KY; ^3^Department of Medical Oncology, Hillman Cancer Center, Pittsburgh, PA, United States; ^4^Department of Surgical Oncology, Hillman Cancer Center, Pittsburgh, PA, United States

**Keywords:** pancreatic adenocarcinoma, stereotactic body radiation therapy, overall survival, local control, fractionation, toxicity

## Abstract

**Background/introduction:**

Early reports of stereotactic body radiation therapy (SBRT) for pancreatic ductal adenocarcinoma (PDAC) used single fraction, but eventually shifted to multifraction regimens. We conducted a single institution review of our patients treated with single- or multifraction SBRT to determine whether any outcome differences existed.

**Methods and materials:**

Patients treated with SBRT in any setting for PDAC at our facility were included, from 2004 to 2014. Overall survival (OS), local control (LC), regional control (RC), distant metastasis (DM), and late grade 3 or greater radiation toxicities from the time of SBRT were calculated using Kaplan–Meier estimation to either the date of last follow-up/death or local/regional/distant failure.

**Results:**

We identified 289 patients (291 lesions) with pathologically confirmed PDAC. Median age was 69 (range, 33–90) years. Median gross tumor volume was 12.3 (8.6–21.3) cm^3^ and planning target volume 17.9 (12–27) cm^3^. Single fraction was used in 90 (30.9%) and multifraction in 201 (69.1%) lesions. At a median follow-up of 17.3 months (IQR 10.1–29.3 months), the median survival for the entire cohort 17.8 months with a 2-year OS of 35.3%. Univariate analysis showed multifraction schemes to have a higher 2-year OS 30.5% vs. 37.5% (*p* = 0.019), it did not hold significance on MVA. Multifractionation schemes were found to have a higher LC on MVA (HR = 0.53, 95% CI, 0.33–0.85, *p* = 0.009). At 2 years, late grade 3+ toxicity was 2.5%. Post-SBRT CA19-9 was found on MVA to be a prognostic factor for OS (HR = 1.01, 95% CI, 1.01–1.01, *p* = 0.009), RC (HR = 1.01, 95% CI 1.01–1.01, *p* = 0.02), and DM (HR = 1.01, 95% CI, 1.01–1.01, *p* = 0.001).

**Conclusion:**

Our single institution retrospective review is the largest to date comparing single and multifraction SBRT and the first to show multifraction regimen SBRT to have a higher LC than single fractionation. Additionally, we show low rates of severe late toxicity with SBRT.

## Introduction

Pancreatic carcinoma is an aggressive malignancy with a predicted 53,070 new cases and 41,780 deaths in the US in 2016 with a 5-year survival of only 7% (1). Currently, tumor resectability is the single most powerful prognostic factor with surgery allowing the only practical chance for cure (2). Systemic chemotherapy and radiation, however, have been shown to play an important role as adjuvant therapy. Unfortunately, less than 20% of patients are deemed surgical candidates at time of presentation due to the commonly advanced presentation of disease (3, 4).

Historically, radiation therapy for pancreatic adenocarcinoma consisted of 6 weeks of external beam radiation therapy (EBRT) which was associated with significant toxicity and delayed time to systemically dosed chemotherapy (5). Drawing upon the success of stereotactic radiosurgery in the treatment of intracranial tumors, stereotactic body radiation therapy (SBRT) was developed in order to deliver a hypofractioned treatment to extracranial tumors (6, 7). Using high precision, this can deliver a high biological effective dose to the tumor while minimizing dose to surrounding tissue (8).

Recently, SBRT has demonstrated utility as primary treatment in unresectable disease, neoadjuvant treatment in locally advanced disease, and adjuvant therapy for resected and recurrent pancreatic tumors (9–14). Due to the shorter duration of treatment in SBRT over standard EBRT, patients receive full dose systemic chemotherapy with less delay. Importantly, studies show SBRT to have excellent local control (LC) and minimal toxicity rates while remaining a cost-effective treatment option (15–17). Initial experience with SBRT for pancreatic adenocarcinoma primarily used single fraction regimens however were complicated by relatively high rates of late GI toxicity (11, 18, 19). Later experiences used multifraction regimens were implemented and have since been shown to limit late GI toxicity (20). Data comparing single- to multifraction regimens, however remain limited and are unclear whether there are any other differences in these treatments besides toxicity rates. We therefore aim to compare single to multifraction SBRT for pancreatic cancer to distinguish possible differences in control rates, overall survival (OS), and toxicity.

## Materials and Methods

### Patient Population

Following approval from our institutional review board, we reviewed patients with histologically proven pancreatic adenocarcinoma treated with SBRT in either one or three fractions between 2004 and 2014. Patients received SBRT as neoadjuvant, adjuvant, or definitive treatment. Patients with resectable, borderline resectable, unresectable, medically inoperable, and recurrent tumors were included in this study. Patients with distant metastasis (DM) at diagnosis (who were provided SBRT for symptom control) were excluded as well as one additional patient excluded for having no records other than SBRT date and dose. Patients included in the study were staged clinically, with the use of CT scans and endoscopic ultrasound techniques. SBRT was performed on either a CyberKnife robotic radiosurgery (Accuray Inc., Sunnyvale, CA, USA) or non-robotic linear accelerator based platforms (Trilogy, TrueBeam) (Varian Medical Systems, Palo Alto, CA, USA). Patient variables including age, race, gender, SMAD4 mutation, surgical status, chemotherapy treatment, prior EBRT, and SBRT dose, dosimetry, and advanced toxicities were collected.

### Definition of Parameters

**Figure 1:**
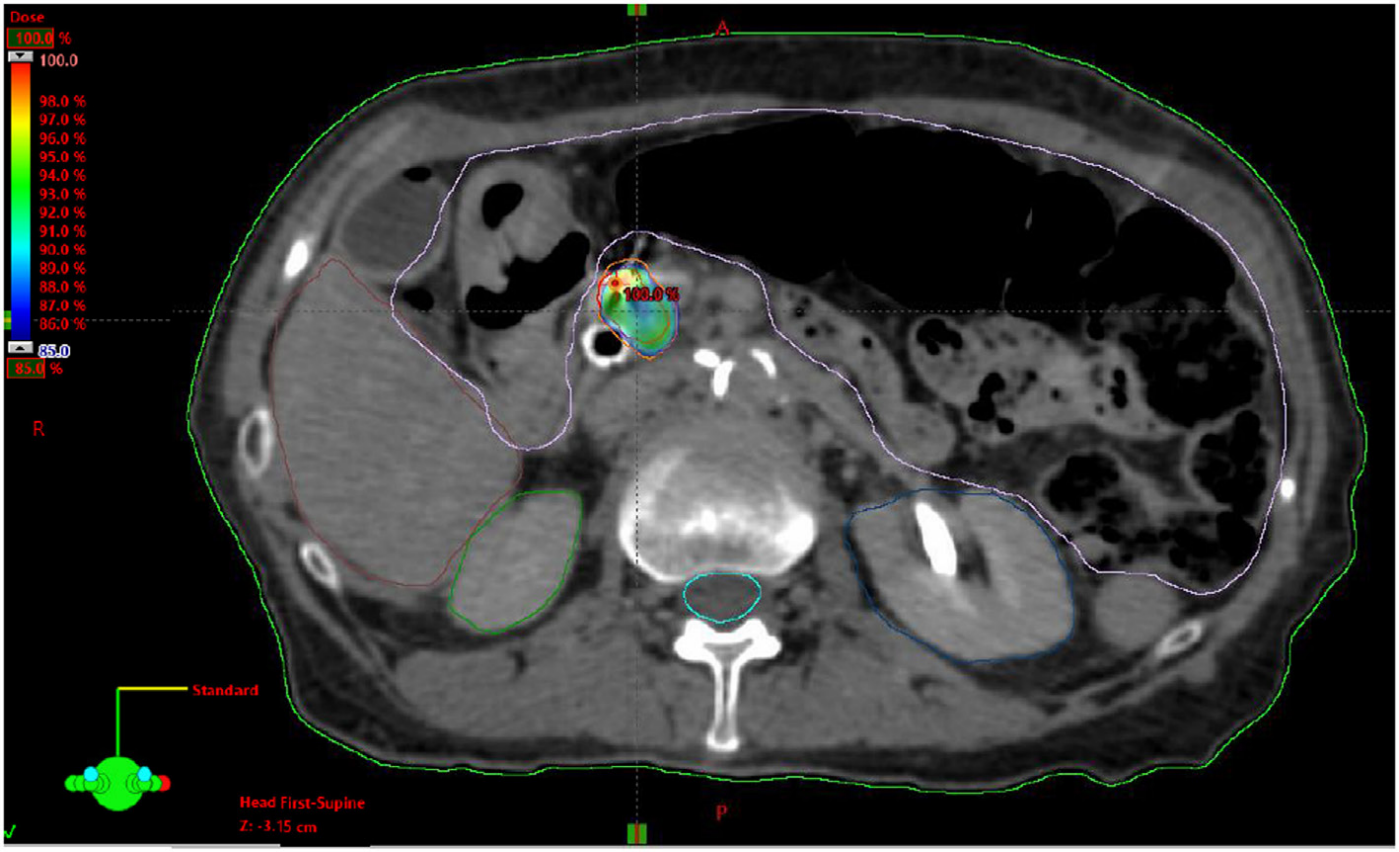
Example stereotactic body radiation therapy plan for pancreatic adenocarcinoma with gross tumor volume highlighted in red, planning target volume highlighted in orange and 85% isodose highlighted in dose heatmap.

Resectable status was determined by a multidisciplinary case review using NCCN guidelines for resectable, borderline resectable, and unresectable disease. Local, regional, and distant progression were determined based on radiographic findings on follow up and/or confirmatory biopsy if done. Local progression was identified as progressive disease using RECIST 1.1 criteria. This is characterized by at least a 20% increase in the sum of diameters of the tumor with minimum of a 5 mm increase (21). Regional failure was defined as disease progression to the regional nodes defined as n1, n2, or n3 by the JPS classification (22, 23) (or new tumor growth within the pancreas outside of the radiation field). Toxicity was graded retroactively with the Common Terminology Criteria for Adverse Events Version 4.0 (CTCAE 4.0). Patients included in this review were simulated in the supine position using four-dimensional CT scan with IV contrast in a vacuum lock bag and wingboard. The 4D-CT scan was obtained utilizing 1.25 mm slices simulated in a vacuum lock bag. During the time of simulation, a motion study was performed during which we obtained multiple images during the respiratory cycle using the abdominal marker as a surrogate for the respiratory cycle. The signal detected from the abdominal surrogate was used to bin the CT images, creating a series of separate CT scans for each phase in the breathing cycle. We then contoured the gross tumor volume (GTV) to see whether any motion was detected during the breathing cycles. If the motion was found to be more than 5 mm, we decided to use respiratory gating. In this technique, we determined which phases of the breathing cycle limit the tumor motion to 5 mm and treat during those specific phases. During the patient’s treatment, an equivalent abdominal surrogate signal is used to control the beam on time of the linear accelerator. The GTV was determined based on the simulation CT scan and diagnostic CT scans. The planning target volume (PTV) margin was added to be approximately 3 mm from GTV with editing off of the bowel (Figure 1). Patients included in the study had fiducials placed before CT-simulation to assist with target delineation during treatment. The bowel was our major dose limiting structured and was limited to no more than 30 Gy maximum. The max dose for the kidneys, liver, and cord were limited to 15, 50, and 15 Gy, respectively. Notably two patients exceeded the max dose for the right kidney (29.0 and 18.3 Gy), and one patient exceeded the max dose for the cord (27.6 Gy).

### Statistical Analysis

Overall survival, LC, regional control (RC), DM, and advanced grade 3 or greater radiation toxicities from the time of SBRT were calculated using Kaplan–Meier estimation to either the date of last follow-up/death or local/regional/distant failure. Predictive factors for OS, LC, RC, DM, and advanced toxicities were determined through use of univariate log-rank test or Cox regression analysis. Those variables deemed significant (*p* < 0.05) were incorporated into multivariable survival analyses using forward stepwise selection in a Cox proportional hazards regression model. Statistical significance was set with a two-sided *p*-value of < 0.05. Statistical analysis was performed using IBM SPSS Statistics version 23.

## Results

### Patient Characteristics

A detailed list of patient characteristics can be found in Table 1. We identified 289 patients with 291 lesions treated with SBRT of pancreatic adenocarcinoma with a median age at diagnosis of 69 (range 33–90) with 49.8% female and 50.2% male. Tumors were located in the head (62.9%), body (11%), uncinate process (7.6%), neck (4.8%), tail (2.7%), and genu (0.3%) of the pancreas. Multifocal disease as defined by two or more lesions within the pancreas was seen in 10.7%. SMAD4 mutation status was collected and positive in 10% and unknown in 78.7% of lesions. Recurrent lesions represented 13.4% of the total and 13.1% of the total lesions received prior EBRT with a median dose of 50.4 Gy in 2–8 fractions (IQR, 30–50.4). Chemotherapy was given to 82.1% of patients and 55.0% received neoadjuvant chemotherapy. Surgical status at diagnosis, deemed in a multidisciplinary case review, included resectable (entire cohort: 39.2%, single fraction: 41.3%, multifraction: 37.7%), borderline resectable (entire cohort: 14.0%, single fraction: 2.2%, multifraction: 19.1%), locally advanced/unresectable (entire cohort: 42.3%, single fraction: 55.4%, multifraction:37.2%), and medically inoperable (entire cohort:4.5%, single fraction: 1.1%, multifraction: 6.0). Surgical resection was performed on 46.7, 44.6, and 47.7% of the entire cohort, single fraction patients, and multifraction patients, respectively.

**Table 1:** Patient characteristics.

Characteristics	Value (*n* = 291 lesions)
**Age (years, range)**	69 (33–90)
**Gender**	
Female	145 (49.8%)
Male	146 (50.2%)

**CA19-9 value (median value, IQR)**	
At diagnosis	221(80–733)
Pre-SBRT	81 (21–378)
Post-SBRT	73 (23–342)

**SMAD4 mutated**	
No	33 (11.3%)
Yes	29 (10%)
Unknown	229 (78.7%)

**Surgical status**	
Resectable entire cohort	112 (39.2%)
Resectable single fraction	38 (41.3%)^a^
Resectable multifraction	75 (37.6%)^b^
Borderline resectable	40 (14.0%)
Borderline resectable single fraction	2 (2.2%)^a^
Borderline resectable multifraction	38 (19.1%)^b^
Unresectable	121 (41.5%)
Unresectable single fraction	51 (55.4%)^a^
Unresectable multifraction	74 (37.2%)^b^
Medically inoperable	13 (4.5%)
Medically inoperable single fraction	1 (1.1%)^a^
Medically inoperable multifraction	12 (6.0%)^b^

**Surgery**	
Yes entire cohort	136 (46.7%)
Yes single fraction	41 (44.6%)^a^
Yes multifraction	95 (47.7%)^b^
No entire cohort	155 (53.3%)
No single fraction	51 (55.4%)^a^
No multifraction	105 (52.3%)^b^

**Any chemotherapy**	
Yes	239 (82.1%)
No	42 (14.4%)
Unknown	10 (3.4%)

**Neoadjuvant chemotherapy**	
Yes	160 (55%)
No	115 (39.5%)
Unknown	16 (5.5%)

**Location**	
Body	32 (11%)
Heal	183 (62.9%)
Tail	8 (2.7%)
Uncinate	22 (7.6%)
Neck	14 (4.8%)
Genu	1 (0.3%)
Multiple	31 (10.7%)

**Prior EBRT**	
Yes	38 (13.1%)
No	253 (86.9%)
Previous EBRT dose (median, IQR)	50.4 (30–50.4)

**Treatment platform**	
Trilogy	120 (41.2%)
CyberKnife	72 (24.7%)
Truebeam	99 (34%)

**Recurrent lesion**	
Yes	39 (13.4%)
No	252 (86.6%)

**GTV (cm^3^) (median, IQR)**	**12.3 (8.6–21.3)**
**PTV (cm^3^) (median, IQR)**	**17.9 (12–27)**

**Fractionation**	
Single	90 (30.9%)
Multifraction	201 (69.1%)

**Dose**	
18 Gy in 1 fraction	2 (0.7%)
20 Gy in 1 fraction	4 (1.4%)
20 Gy in 2 fractions	1 (0.3%)
22 Gy in 1 fraction	17 (5.8%)
24 Gy in 1 fraction	62 (21.3%)
24 Gy in 2 fraction	1 (0.3%)
24 Gy in 3 fraction	2 (0.7%)
25 Gy in 1 fraction	5 (1.7%)
27 Gy in 3 fractions	7 (2.4%)
30 Gy in 3 fractions	28 (9.6%)
36 Gy in 3 fractions	162 (55.7%)

**BED_10 Gy_**	
Median (IQ range)	180 Gy_10_ (180–183.3)

*^a^Percentages out of the 92 patients who received single-fraction SBRT*.

*^b^Percentages out of the 199 patients who received multifraction SBRT*.

*SBRT, stereotactic body radiation therapy*.

### SBRT Treatment Characteristics

Stereotactic body radiation therapy was delivered by Trilogy (41.2%), Truebeam (34.1%), or CyberKnife (24.7%) in either one fraction (30.9%) or multiple fractions (69.1%). Median dose was 24 Gy (range 18–25) for single fraction and 36 Gy (range 24–36) for three fractions. One patient received 24 Gy in two fractions with all others receiving three-fraction regimens if treated in multiple fractions. For the entire cohort, GTV was 12.3 cm^3^ (IQR 8.6–21.3) and PTV was 17.9 cm^3^ (IQR 12–17). For patients who received neoadjuvant SBRT, the median time to surgery was 1.8 months (IQR 1.44–3.84). For those who received adjuvant SBRT, the median time from surgery was 3.77 months (IQR 2.17–12.15).

### Overall Survival

At a median follow-up of 17.3 months (IQR 10.1–29.3 months), the median survival for the entire cohort was 17.8 months with a 2-year OS of 35.3% (Table 2). Univariate analysis demonstrated superior 2-year survival was significantly associated with age (*p* < 0.001), pre-SBRT CA19-9 (*p* < 0.001), post-SBRT CA19-9 (*p* = 0.011), non-robotic treatment platform (*p* = 0.013), PTV volume (*p* < 0.001), recurrent lesions (*p* = 0.001), surgery (*p* < 0.001), and multifraction SBRT (*p* = 0.019). While univariate analysis showed multifraction schemes to have a higher 2-year OS 30.5% vs. 37.5%, it did not hold significance on multivariate analysis. Only surgery [*p* = < 0.001, HR 0.31 (95% CI, 0.19–0.51)] and post-SBRT CA19-9 [*p* = 0.009, HR 1.01 (95% CI, 1.01–1.01)] maintained significance on multivariate analysis (Table 3). For patients receiving resection, 2-year OS was 59.8% compared to 14.3% for those not receiving surgical resection.

**Table 2:** Univariate and multivariate analyses for overall survival (OS).

Variable	2-year OS	*p* Value
**Age**	**–**	**<0.001**

**CA19-9**		
At diagnosis	–	0.604
Pre-SBRT	–	**<0.001**
By median (≤83 vs. <83)	–	0.457
≤90 vs. >90	–	0.347
Post-SBRT	–	**0.011**
By median (≤73 vs. >73)	54.1 vs. 26.4%	**<0.001**
≤90 vs. >90	52.1 vs. 25.5%	**<0.001**
SMAD4 mutation		0.968
Location	–	0.283
Prior EBRT	–	0.241

**Treatment platform**		
All (Trilogy, Truebeam, CK)	39.5 vs. 27.8 vs. 35.9%	**0.036**
Non-robotic (T/T) vs. CyberKnife	37.9 vs. 27.8%	**0.013**
All (Trilogy, Truebeam, CK)		

**Recurrent lesion**		
Recurrent vs. not	69.4 vs. 30.4%	**0.001**
Surgery (yes vs. no)	**59.8 vs. 14.3%**	**<0.001**

**Dosimetry**		
GTV volume	–	0.399
GTV max dose	–	0.163
GTV min dose	–	0.793
PTV volume	–	**<0.001**
By median (≤18 vs. >18 cm^3^)	44 vs. 31.9%	**0.033**
PTV max dose	–	0.847
PTV min dose	–	0.529
PTV mean dose	–	0.982
Small bowel max dose	–	0.365
Small bowel mean dose	–	0.179
Single vs. multifraction	30.5 vs. 37.5%	**0.019**
BED_10 Gy_	–	0.151

**Variable**	**HR (95% CI)**	***p* Value**

Post-SBRT CA 19-9 (continuous)	1.01 (1.01–1.01)	**0.009**

**Surgery**		
Not completed	1.00 (reference)	–
Completed	0.31 (0.19–0.50)	**<0.001**

*Boldface values are significant predictors (p value < 0.05)*.

**Table 3:** Univariate and multivariate analysis for local control (LC).

Variable	2-year LC	*p* Value
Age	–	0.549

**CA 19-9**		
At diagnosis	–	0.581
Pre-SBRT	–	0.820
Post-SBRT	–	0.082
SMAD4 mutation	–	0.791
Location	–	0.427
Prior EBRT	–	0.586

**Treatment platform**		
All (Trilogy, Truebeam, CK)	73.7 vs. 61.7 vs. 58.5%	**0.022**
Non-robotic (T/T) vs. CyberKnife	68.2 vs. 58.5%	**0.010**
All (Trilogy, Truebeam, CK)		

**Recurrent lesion**		
Recurrent vs. not	35.4 vs. 70.4%	**<0.001**
Surgery (yes vs. no)	74.1 vs. 53.9%	**0.010**

**Dosimetry**		
GTV volume	–	0.419
GTV max dose	–	0.145
GTV min dose	–	0.223
PTV volume	–	0.260
PTV max dose	–	0.196
PTV min dose	–	0.753
PTV mean dose	–	0.213
Small bowel max dose	–	0.657
Small bowel mean dose	–	0.355
Single vs. multifraction	56.8 vs. 69.7%	**0.004**
BED_10 Gy_ (continuous)	–	0.052

**Variable**	**HR (95% CI)**	***p* Value**

**Recurrent lesion**		
No	1.00 (reference)	**0.003**
Yes	2.31 (1.32–4.05)	

**Fractionation scheme**		
Single fraction	1.00 (reference)	**0.009**
Multifraction	0.53 (0.33–0.85)	

*Boldface values are significant predictors (p value < 0.05)*.

### Local Control

**Figure 2:**
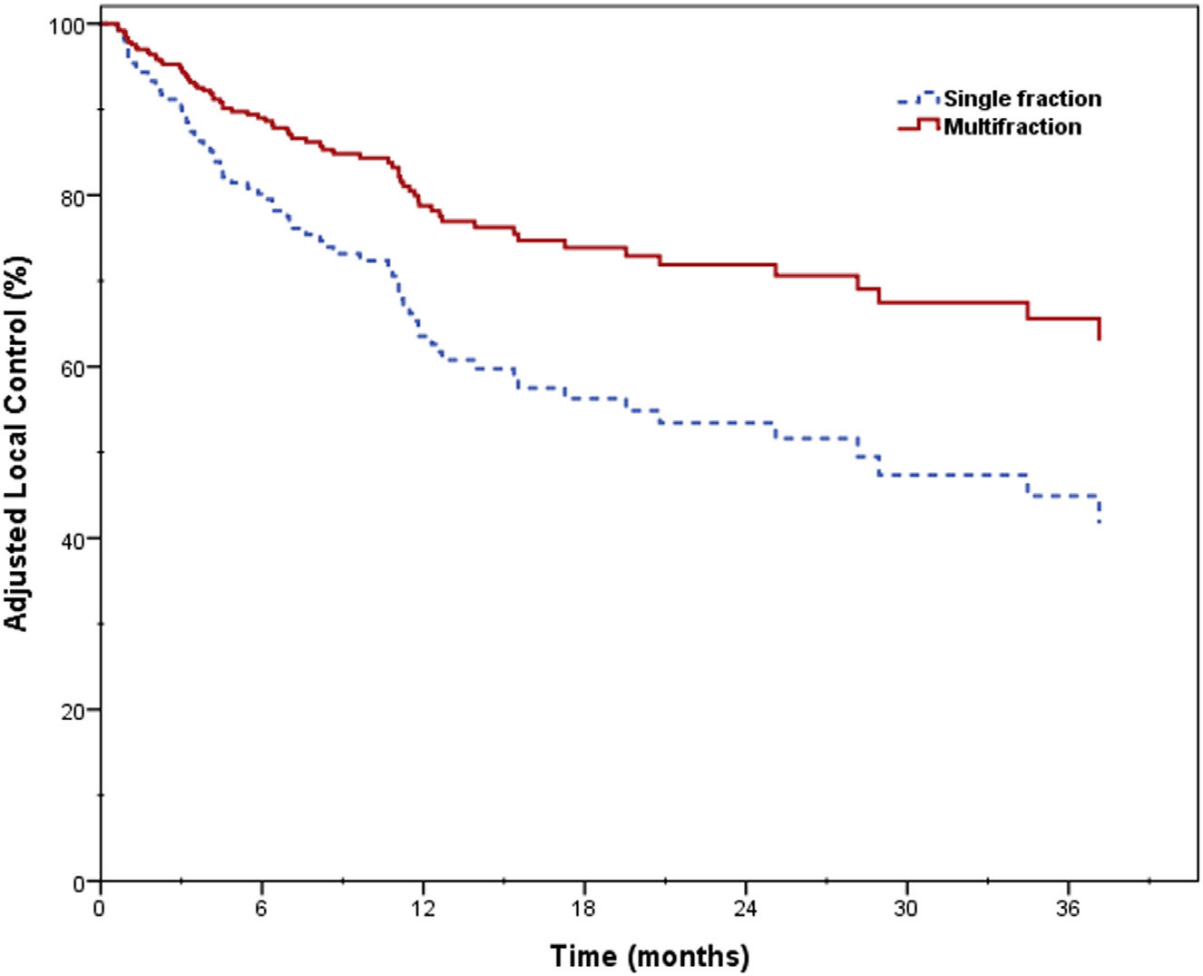
Local control for single- and multifraction stereotactic body radiation therapy.

Two-year LC was 66.1% for the entire cohort, 56.8% for single fraction, and 69.7% for multifraction SBRT (Figure 2). Univariate analysis demonstrated superior 2-year LC significantly associated with treatment platform (*p* = 0.002), non-recurrent lesions (*p* < 0.001), surgery (*p* = 0.01), and multifraction SBRT (*p* = 0.004). Two-year LC for patients who received surgical resection was 74.1 vs. 53.9% for those who did not. On multivariate analysis, multifractionation [*p* = 0.009, HR 0.53 (95% CI, 0.33–0.85)] was associated with higher LC, whereas recurrent lesions led to lower LC [*p* = 0.003, HR 2.31 (95% CI, 1.32–4.05)] (Table 4). Two-year LC for recurrent lesions was 35.4% compared to 70.4% for non-recurrent lesions.

**Table 4:** Univariate and multivariate analysis for regional control (RC).

Variable	2-year RC	*p* Value
Age	–	0.271

**CA 19-9**		
At diagnosis	–	0.535
Pre-SBRT	–	0.413
Post-SBRT	–	**0.020**
By median (≤73 vs. >73)	–	0.580
≤90 vs. >90	–	0.891
SMAD4 mutation	–	0.677
Location	–	0.942
Prior EBRT	–	0.677

**Treatment platform**		
All (Trilogy, Truebeam, CK)	–	0.735
Non-robotic (T/T) vs. CyberKnife	–	0.725

**Recurrent lesion**		
Recurrent vs. not	–	0.137
Surgery (yes vs. no)	–	0.434

**Dosimetry**		
GTV volume	–	0.685
GTV max dose	–	0.904
GTV min dose	–	0.761
PTV volume	–	0.752
PTV max dose	–	0.958
PTV min dose	–	0.608
PTV mean dose	–	0.789
Small bowel max dose	–	0.748
Small bowel mean dose	–	0.600
Single vs. multifraction	–	0.541
BED_10 Gy_ (continuous)	–	0.067

**Variable**	**HR (95% CI)**	***p* Value**

Post-SBRT CA 19-9	1.01 (1.01–1.01)	**0.020**

*Boldface values are significant predictors (p value < 0.05)*.

### RC and Distant Metastases

One- and two-year RC rates were 89.2 and 86.3%, respectively. A higher post-SBRT CA19-9 was the only variable found to be significantly associated with inferior RC on univariate and multivariate analysis [*p* = 0.02, HR 1.01 (95% CI, 1.01–1.01)] (Table 5). At one and two years, the Kaplan–Meier estimated rate of DM was 39.5 and 56.4%, respectively. Univariate analysis identified CA19-9 at diagnosis (*p* = 0.041), pre-SBRT CA 19-9 (*p* = 0.001), and post-SBRT CA 19-9 (*p* = 0.006) associated with increased distant metastases. Post-SBRT CA19-9 was the only variable to maintain significance on multivariate analysis [*p* = 0.001, HR 1.01 (95% CI, 1.01–1.01)] (Table 5). Treatment fractionation was not found to be associated with either RC or distant metastases.

**Table 5:** Univariate and multivariate analysis for distant metastasis (DM).

Variable	2-year DM	*p* Value
Age	–	0.348

**CA 19-9**		
At diagnosis	–	**0.041**
Pre-SBRT	–	**0.001**
By median (≤83 vs. >83)	48.9 vs. 59.8%	**0.039**
≤ 90 vs. >90	50.3 vs. 59.2%	0.054
Post-SBRT	–	**0.006**
By median (≤73 vs. >73)	41.8 vs. 70.8%	**0.004**
≤90 vs. >90	47.7 vs. 66.6%	0.067
SMAD4 mutation	–	0.696
Location	–	0.745
Prior EBRT	–	0.815

**Treatment platform**		
All (Trilogy, Truebeam, CK)	–	0.467
Non-robotic (T/T) vs. CyberKnife	–	0.163
Recurrent lesion		
Recurrent vs. not	–	0.586
Surgery (yes vs. no)	–	0.124

**Dosimetry**		
GTV volume	–	0.664
GTV max dose	–	0.380
GTV min dose	–	0.694
PTV volume	–	0.564
PTV max dose	–	0.480
PTV min dose	–	0.176
PTV mean dose	–	0.900
Small bowel max dose	–	0.487
Small bowel mean dose	–	0.499
Single vs. multifraction	–	0.226
BED_10 Gy_ (continuous)	–	0.429

**Variable**	**HR (95% CI)**	***p* Value**

Post-SBRT CA 19-9	1.01 (1.01–1.01)	**0.001**

*Boldface values are significant predictors (p value < 0.05)*.

### Late Radiation Toxicity

For the entire cohort, the Kaplan–Meier estimated advanced grade 3+ toxicity rate at 1- and 2-years was 2.5% (95% CI, 2.3–2.7%) advanced grade. No significant difference was noted based on single- or multifraction use (2-year advanced grade 3+ toxicity estimate 2.3 vs. 2.8%, *p* = 0.747). Prior radiation therapy was the only factor predictive of advanced grade 3+ toxicity [*p* = 0.019, HR 4.58 (95% CI, 1.29–16.21)] (Table 6). For patients who received surgery, advanced grade 3+ toxicity rate at 2-years was 1.8% compared to 3.2% for non-surgical patients (*p* = 0.258). Three patients experienced grade 4 toxicities which included an ileal obstruction (*n* = 1), obstruction gastric (*n* = 1), and duodenal stenosis (*n* = 1) all requiring urgent operative intervention. Eight patients experienced grade 3 toxicities which included nausea (*n* = 3), enteritis (*n* = 2), enterocolitis (*n* = 1), ileal hemorrhage (*n* = 1), and biliary tract infection (*n* = 1).

**Table 6:** Kaplan–Meier estimates for various end points.

End point	Kaplan–Meier estimate (95% confidence interval)	
**Median follow-up**		
From SBRT (months) (IQ range)	17.3 (10.1–29.3)	

**Median survival**		
Median survival (months) (95% CI)	17.8 (15.7–20.0)	
12 months (95% CI)	69.7 (64.4–75.0)	
24 months (95% CI)	35.3 (29.8–40.9)	

**Local control from SBRT**		
12 months (95% CI)	73.7 (67.6–79.8)	
24 months (95% CI)	66.1 (58.5–73.4)	

**Regional control from SBRT**		
12 months (95% CI)	89.2 (84.9–93.5)	
24 months (95% CI)	86.3 (81.0–91.6)	

**Distant metastases from SBRT**		
12 months (95% CI)	39.5 (33.0–46.0)	
24 months (95% CI)	56.4 (48.6–64.2)	

**Toxicity**		
Grade 2+ toxicity		
1-year toxicity (95% CI)	7.8 (4.7–10.9)	
2-year toxicity (95% CI)	7.8 (4.7–10.9)	
Grade 3+ toxicity		
1-year toxicity (95% CI)	2.5 (2.3–2.7)	
2-year toxicity (95% CI)	2.5 (2.3–2.7)	

**Univariable analysis for advanced toxicities**		

**Variable (grade 2+advanced toxicity)**	**HR (95% CI)**	***p* Value**

Prior radiotherapy	3.26 (1.33–8.00)	0.010
GTV volume	–	0.201
PTV volume	–	0.736
Treatment platform	–	0.57
Single vs. multifraction	–	0.737
Small bowel max dose	–	0.955
Small bowel mean dose	–	0.774

**Variable (grade 3+ advanced toxicity)**	**HR (95% CI)**	***p* Value**

Prior radiotherapy	4.58 (1.29–16.21)	0.019
GTV volume	–	0.396
PTV volume	–	0.997
Treatment platform	–	0.427
Single vs. multifraction	–	0.983
Small bowel max dose	–	0.899
Small bowel mean dose	–	0.795

## Discussion

This retrospective review aimed to assess the role SBRT fractionation on OS and LC of pancreatic adenocarcinoma while also determining the rates of late radiation toxicity for this treatment modality. This study identified three-fraction SBRT provides superior 2-year LC over single fractionation, but may not be associated with improved OS. Additionally, treatment with either single- or multifraction SBRT was delivered with reasonable levels of toxicity with prior radiation therapy most predictive of late radiation toxicities. Consistent with numerous other reports, these data also support surgery as the most important factor for OS (24).

Pollom et al. previously reported on 167 patients with unresectable pancreatic adenocarcinoma comparing single- versus five-fraction SBRT. Median OS for all patients was 13.6 months with 12-month survival from SBRT at 30.8 and 34.9% for single- and multifraction, respectively. The 12-month cumulative incidence rates of local recurrence were 9.5 and 11.7% for single and multifraction, respectively. Neither OS from SBRT nor local recurrence demonstrated significant difference between single- and multifraction groups. Cumulative GI grade 2+ toxicity by 12 months was 26.1% for single fraction and 7.8% for multifraction which was a significant difference (20). In contrast, our data show a significant improvement in LC with multifraction as compared to single-fraction regimens. This difference is most likely due to the significantly larger cohort used in the present study. Additional differences that may have contributed included our study reporting on all pancreatic adenocarcinoma except those with distant metastases as opposed to only unresectable tumors including those with distant metastases at treatment. Additionally, our multifraction group consisted of 36 Gy in three fractions as opposed to 33 Gy in five fractions. Finally, because the linear quadratic model in not applicable for hypofractionated treatments, there could be a difference in BED between these two regimens which is currently unknown.

We believe our difference observed in LC between single and multifraction SBRT is most likely a result of differences in tumor dose. In our initial experience with single fraction SBRT there was significant concern for bowel toxicity. In an effort to reduce toxicity, dose to the small bowel was limited therefore compromising PTV coverage. Multifraction regimens allowed us to be less conservative with bowel dosing and therefore allow for greater coverage of the tumor. This would further explain why we did not observe a difference in advanced grade 3+ toxicity between these regimens as demonstrated by Pollom et al. These two studies seem to demonstrate the trade-off between toxicity and LC for single fraction SBRT. Our observed difference in LC however could also be a result of patient demographics independent of fractionation. As reports of increased toxicity with single fraction surfaced, our institution slowly switched to multifraction regimens. This lead a greater proportion of patients treated in later years received three-fraction SBRT. It is possible this observed difference was a result of differences in chemotherapy; however, chemotherapy regimens were poorly reported and demonstrated significant heterogeneity between and within single- and multifraction cohorts. Additionally, there was higher proportion of recurrent disease in those treated with single fraction SBRT. As we have shown, patients with recurrent disease demonstrated worse LC which may have affected the results observed in single-fraction patients. Although surgical resection has been shown to be the most significant predictive factor for clinical outcomes, it is unlikely playing a role in the observed difference between different fraction regimens as resection rates were similar between both groups. SBRT has also been an enticing modality in the treatment of locally advanced and borderline resectable pancreatic adenocarcinoma due to its improved rates of LC and increased rates of resection in these tumors (25). Although previous trials analyzing single fraction SBRT demonstrated excellent LC, they were associated with unacceptable rates of toxicity (18, 19). This leads to the adoption of multifraction regimens. Chuong et al. reported on 73 patients with borderline resectable (78.1%) and locally advanced (21.9%) pancreatic adenocarcinoma treated with induction chemotherapy followed by five-fraction SBRT. Borderline resectable and locally advanced tumors experienced a median OS of 16.4 and 15 months and a 1-year progression free survival of 42.8 and 41%, respectively, with 5.3% advanced grade 3+ toxicity (10). Mahadevan et al. reported on 36 patients with nonmetastatic locally advanced pancreatic cancer treated with three-fraction SBRT with a total dose of 24, 30, or 36 Gy followed by gemcitabine therapy. Median OS was 14.3 months; LC was 78% with a median progression-free survival of 9.6 months and 5.5% (*n* = 2) developed advanced grade 3+ toxicity. Although previous reports identified unacceptable rates of advanced toxicity with single-fraction SBRT our results demonstrated no significant difference between single- and multifractionation on advanced toxicity, which may reflect variances in volumes, constraints and total dose between institutions. Additionally, as previously stated, due to early reports of high rates of late GI toxicity, our institution limited dose to the small bowel in an attempt to minimize toxicity rates. Our lack of ability to identify differences in toxicity could also be secondary to the inherent limitation of retrospective studies.

Stereotactic body radiation therapy has also been identified as an effective treatment modality in treatment of recurrent lesions following resection. Dagolglu et al. reported on 30 patients treated with SBRT for recurrent pancreatic cancer with prior radiation therapy. Patients received a median of 25 Gy in five fractions. Median OS was 14 months, 2-year LC was 78, and 7% advanced grade 3+ toxicity (26). Here, we reported significantly greater risk of advanced toxicity with prior radiation therapy while Dagolglu et al. demonstrated reasonable toxicity in patients with prior radiation. The lower toxicity seen with Dagolglu et al. could be secondary to the five fraction regimen used as opposed to the three fractions or one fraction mostly used in our study. Also, 25 Gy in five fractions has a lower biological effective dose than our regimens, which may compromise LC.

Herman et al. recently reported on a multi-institutional study combining Gemcitabine with five-fraction SBRT in patients with locally advanced pancreatic cancer (27). Forty-nine patients with locally advanced patients were treated with up to three doses of Gemcitabine (1,000 mg/m^2^) followed by a 1-week break and then SBRT, 33 Gy in five fractions over 1–2 weeks. After SBRT, patients were continued on Gemcitabine until progression. Median OS was 14 months and 2-year OS was 18%. Freedom from local progression at 1-year was 78%. On multivariate analysis PET positive disease prior to SBRT and CA19-9 > 90 after SBRT were associated with an increased risk of death. Acute grade 2 plus toxicity included enteritis, gastritis and ulcer. There was 1 grade 4 toxicity of a duodenal fistula. There was 11% advanced grade 2 or greater toxicity, again mostly enteritis, gastritis, and ulcer. Their advanced toxicity was low like ours showing feasibility of multifraction regimens.

We showed higher post-SBRT CA 19-9 were associated with lower survival and higher rates of regional and DM. This is similar to the results reported by Herman et al, who showed post-SBRT values greater than 90 were associated with a lower survival on multivariate analysis (27). These results of post-SBRT CA19-9 associated with survival but not CA19-9 at diagnosis or pre-SBRT indicate posttreatment CA19-9 as a surrogate for treatment efficacy. Similar data have been reported in the literature identifying a decrease in CA19-9 following treatment to be a predictor of OS (28–30). Future trials evaluating the role of SBRT for pancreatic carcinoma should consider evaluating the affect SBRT has on CA19-9 values.

This study adds to the current growing body of literature that demonstrates the effectiveness and tolerability of SBRT for pancreatic adenocarcinoma. Following the initial reports demonstrating unacceptably high rates of advanced GI toxicity with single fraction SBRT, many institutions transitioned to multifraction regimens. Despite previous reports indicating multifraction SBRT produced decreased rates of toxicity, it was unclear whether this came at the cost of other clinical outcomes. Through analyzing the largest cohort comparing single to multifraction SBRT, we identified multifraction treatment schedules to be associated with improved LC while reinforcing the significant role surgery and CA19-9 levels play in prognosis (2, 18, 31–34). These results support the continued use of multifraction regimens. Taken together with prior work, multifraction SBRT appears to provide either improved LC or reduced rates of toxicity as compared to single fraction (20).

Despite the above study showing a significant difference in LC between SBRT fractionation, there were numerous limitations to this work. Our cohort represented a very heterogeneous population as it included resected and unresected disease, recurrent disease, as well as patients with prior EBRT. Additionally, as this was a retrospective review the breakdown of patients within one- and three-fraction SBRT were not matched. Another limitation was our inability to capture detailed chemotherapy data on these patients. As patients with single fraction were treated in earlier years it is possible there is an unseen effect of advancements in chemotherapy over time. Regarding toxicity, although we report very low rates, this may be artificially low due to uncaptured toxicity associated with retrospective reports. However, our low toxicity rates are possibly secondary to our institution editing the PTV out of the bowel to reduce toxicity. Finally, CA19-9 levels and SMAD4 mutation status were not obtained in all patients, likely due to limited testing. Prospective studies will be needed to provide a more rigorous analysis of the role of SBRT fractionation in pancreatic adenocarcinoma as well as the possible contribution of SMAD4 mutation status and evolving systemic therapy on LC.

## Conclusion

This single institution retrospective review of 291 patients with pancreatic adenocarcinoma identified multifraction regimens SBRT had a higher LC than single fractionation regimens. Although multifraction regimens displayed a higher OS on univariate analysis it did not hold significance on MVA. Post-SBRT CA19-9 was found to be significant factor for OS, RC, and DM. Finally, we showed low rates of advanced grade 2+ and grade 3+ toxicity associated with SBRT. This single institution report is the largest retrospective series showing multifraction regimens SBRT is associated with a higher LC than single fractionation regimens.

## Ethics Statement

The following study was approved by the University of Pittsburgh Institutional Review Board.

## Author Contributions

PS: collected data from electronic medical records and paper records; wrote and revised manuscript; and approves final version of manuscript. MB: conceived and designed the project; assisted with data collection; revised the manuscript multiple times; and approves final version of manuscript. BG: performed statistical analysis on collected data; revised the manuscript; and approves final version of manuscript. KH: collected data regarding SMAD4 mutation status; provided revisions to manuscript; and approves final version of manuscript. KQ: aided in the interpretation of results as they related to radiation oncology; provided revisions to manuscript; and approves final version of manuscript. NB: aided in the interpretation of results as they relate to medical oncology; provided revisions to manuscript; and approves final version of manuscript. SB: aided in the interpretation of results as they relate to radiation oncology; provided revisions to manuscript; and approves final version of manuscript. HZ: aided in the interpretation of results as they relate to surgical oncology; provided revisions to manuscript; approves final version of manuscript. DH: conceived and designed project with MB; provided multiple revisions to manuscript; and approves final version of manuscript.

## Conflict of Interest Statement

The authors declare that the research was conducted in the absence of any commercial or financial relationships that could be construed as a potential conflict of interest.
